# Application of UAV in Topographic Modelling and Structural Geological Mapping of Quarries and Their Surroundings—Delineation of Fault-Bordered Raw Material Reserves

**DOI:** 10.3390/s20020489

**Published:** 2020-01-15

**Authors:** Ákos Török, Gyula Bögöly, Árpád Somogyi, Tamás Lovas

**Affiliations:** 1 Department of Engineering Geology and Geotechnics, Budapest University of Technology and Economics, H-1111 Budapest, Hungary; bogoly.gyula@epito.bme.hu; 2 Department of Photogrammetry and Geoinformatics, Budapest University of Technology and Economics, H-1111 Budapest, Hungary; somogyi.arpad@epito.bme.hu (Á.S.); lovas.tamas@epito.bme.hu (T.L.)

**Keywords:** UAV, surface model, fault zone, spatial analysis, limestone reserve

## Abstract

A 3D surface model of an active limestone quarry and a vegetation-covered plateau was created using unmanned aerial vehicle (UAV) technique in combination with terrestrial laser scanning (TLS). The aim of the research was to identify major fault zones that dissect the inaccessible quarry faces and to prepare a model that shows the location of these fault zones at the entire study area. An additional purpose was to calculate reserves of the four identified lithological units. It was only possible to measure faults at the lowermost two meters of the quarry faces. At the upper parts of the quarry and on the vegetation-covered plateau where no field geological information was available, remote sensing was used. Former logs of core drillings were obtained for the modelling of the spatial distribution of four lithological units representing cover beds and various quality of limestone reserves. With the comparison of core data, field measurements and remote sensing, it was possible to depict major faults. Waste material volumes and limestone reserves were calculated for five blocks that are surrounded by these faults. The paper demonstrates that, with remote sensing and with localised control field measurements, it is possible: (a) to provide all geometric data of faults and (b) to create a 3D model with fault planes even at no exposure or at hardly accessible areas. The surface model with detected faults serves as a basis for calculating geological reserves.

## 1. Introduction

Recent technological developments of unmanned aerial systems (UAS), including unmanned aerial vehicles (UAV) and data processing technologies, lead to extensive use of these techniques in various fields [1,2,3]. Remotely piloted systems are able to support numerous mapping applications, including forestry [4], archaeology and heritage sites [5,6], or emergency mapping [7]. UAS technology allows the application of multiple sensors [8]. The advanced navigation potential of UAVs allows surveying the extreme topography in a near-vertical road cut-slope [9]. The application of UAS together with terrestrial laser scanning (TLS) enables support for forensic engineering where surveys must be executed in a short time while collecting the greatest possible amount of information with high accuracy [10]. He et al. [11] estimated the stockpile volumes carried on barges using UAS without ground control points. Combined laser scanned and fixed-wing UAS point clouds were used for flood volume and area estimation [12]. The UAV technique itself is capable of detecting rapid changes in the morphology of rivers [13,14] and erosion processes [15], or stream restoration [16]. Ong et al. [17] used UAV and GPS to map floating covers in wastewater treatment plants and evaluated the strain level and health of the structures. UAV was also used for the environmental modelling of an open-pit gravel mine and for measuring the volumetric change [18]. Buildings and morphological changes after earthquakes can be surveyed by UAV-based technique [19] as well, and the data acquisition could also be supported by terrestrial laser scanning [20]. The investigations showcased the potential of the combined technologies in surveying vertical structures and complex buildings with high accuracy. Obviously, along with the data acquisition technologies, the data-processing techniques are also developing at a remarkable speed. The method is also used in agriculture. For example, Comba et al. [21] presented an unsupervised algorithm for vineyard detection based on 3D point-clouds captured by UAS. Volcanological research can be effectively supported by UAS as well by enabling the survey of areas with limited accessibility [22]. Other examples to show the potential of UAS technology and laser scanning in capturing the geometry of inaccessible or hazardous environments are: monitoring ice sheets [23] and ice covered areas where ground control points cannot be used [24]; rapid melting dynamics of glaciers [25]; detection of ice dynamics and snow melts [23,26,27].

This technology effectively supports engineering geological and geotechnical applications [28]. One of the most common applications is the assessment of slope stability [29,30,31,32] and geohazards [30]. Gathering data for stability assessment includes imaging slope profiles and block size [33] and detecting possible discontinuity surfaces that can contribute to slope instability. Previous work has demonstrated that the application of UAV is a feasible and rapid method to obtain slope geometry data and morphological changes [23,29,34]. Slope profiles can be obtained from various sources of point clouds [35,36]. In the stability analysis of cliff faces, the identification of joint systems and fractures are key issues. The remote sensing techniques are capable of identifying these structural geological elements [30,37]. Novel methods and close range surveys using digital techniques include the use of thermo-camera [38], close-range photography [34,39], or TLS [40]. However, at many of these sites, steep cliffs have limited access, and on-site measurements are partly or entirely impossible due to rockfall hazard. This is where the application of remote sensing techniques demonstrates many advantages. In the assessment of the structural geological conditions, these tools are often applied [29,41] for representing faults [37,42] or folds [43].

Several studies have demonstrated that the UAV-based survey and processing could be especially beneficial to the mining industry, especially in open pit mining [44]. UAV systems with the combination of TLS also brought new results. In the cited study, the surface models derived from the different surveys are compared. Remote sensing technique can also be used for reserve calculation [29]. High-resolution radiation maps of a uranium mine by UAV-based radiometric survey is also possible [38]. Recultivated, former mining areas have also been mapped using this technique [45].

In many previous engineering geological studies, remote sensing techniques were focused on supporting slope stability and rockfall hazard assessment by surveying the area of interest with UAV and TLS [29,30,32,46,47,48]. To the contrary, in this paper, the data obtained using these techniques are used for geological reserve calculations. Reserves are crucial in quarry operations for appropriate planning, especially when the deposit is fractured [33,37]. The identification of fractures is possible in subsurface quarries by using TLS [33] and on open rock faces by applying TLS or UAV [37]

The current research focuses on the combined application of UAV and TLS in surface quarrying activities. The study deals with an area where active quarry and potential limestone reserves are found. The limestone reserve is not homogenous; it has several different lithological units representing different stone qualities (from high-quality pure limestone to dolomitic limestone) and has cover beds (overburden) that are considered as waste material. The active quarry exposes steep vertical faces that are hardly accessible, and the technique was used to identify fault zones and major structural units. The terrestrial laser scanner survey was also used as a complementary technique to visualise the quarry faces. Structural geological features (bedding planes, faults) were also measured for reference at accessible parts of the quarry. There were areas where no on-site structural geological measurements were possible, such as steep quarry faces or vegetation covered plateau with no outcrops. The main aim of the application of combined techniques was to create a geometric model of the exploration area and to identify major fault zones that form borderlines between exploitable limestone and dolomitic limestone units as well as clayey cover beds. This allowed for the delineation of limestone reserve and the prediction of faults at areas where no direct structural geological information was available. Overall, the paper intends to demonstrate the applicability of the UAV and TLS technique in the structural geological survey of inaccessible or vegetation-covered areas and its use in geological reserve calculations.

## 2. Study Area

**Figure 1:**
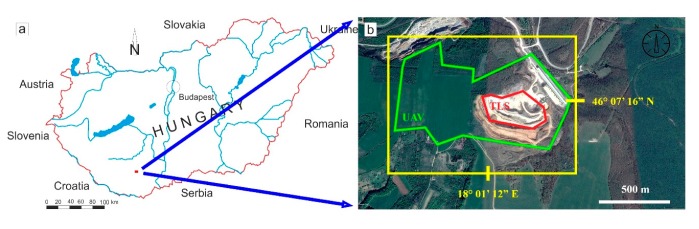
Location of the study site: (**a**) Hungary with the study site (**b**) close view of the study area with WGS 84 coordinates and unmanned aerial vehicle UAV (green boundary line) and TLS (red boundary line) surveyed areas (Google Earth).

**Figure 2:**
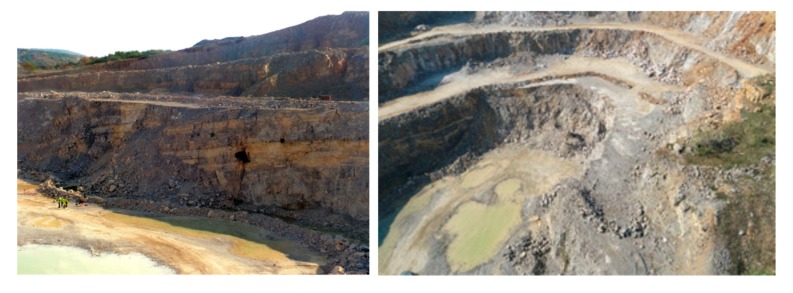
Exposed Middle Triassic carbonates in the operating quarry (left: view from NE, right: image captured from the UAV).

**Figure 3:**
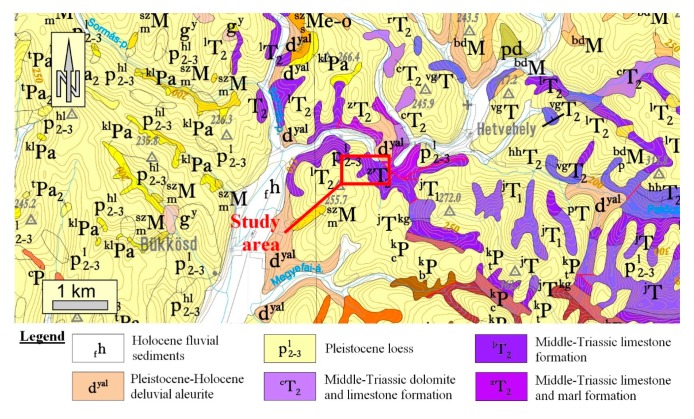
Geological map of the study area and its surroundings. The surveyed area is characterised by Middle Triassic carbonates and younger coverbeds [49].

The study area is located in southern Hungary (Figure 1a) in the Mecsek Mountains. The Mecsek Mountains form a part of the Tisza Mega-unit, a large structural unit that is mostly characterised by Permian-Cretaceous sedimentary sequences with Tertiary and Quaternary cover beds [45]. The area has relatively low relief and high vegetation cover, which makes it challenging to study the geological conditions. The only locations where rocks are exposed are cliff faces of former and currently operated quarries. The study area represents two very different geomorphologic areas; a plateau (Figure 1b) and an operating quarry (Figure 2). These geomorphological differences invoked the division of the surveyed area into two parts. Hence, the UAV flights were made in two steps. The current paper deals with the structural geological aspects of a Triassic carbonate sequence and its cover beds that are exposed in the Western part of the Mecsek Mountains (Figure 1). It is typical cyclic carbonate sediment that was deposited on a homoclinal ramp [39] in the Peri-Tethyan realm, the so-called “Germanic Basin”. The sequence is analogous to Germano-type Triassic carbonates of Poland, Germany [40] and Bulgaria [41]. Lithology is dominated by bioturbated, marly limestones, nodular limestones, homogeneous micritic limestone, with bioclastic limestone intercalation [39]. Yellow, clayey or slightly dolomitic limestone and dark grey organic-rich limestone intercalations also occur. From a structural geological point of view, the study area forms a part of the western Mecsek anticline system. Besides minor folding, the Triassic carbonates are dissected by NE-SW faults according to the geological map (Figure 3). The quarry exposes the Triassic limestone sequence in four levels. The dimensions of the operation quarry are 550 × 420 m. However, the present study also deals with the reserve area with future quarrying (Figure 1).

## 3. Methods

The study methods can be divided into three parts: (1) survey with TLS and UAV and preparation of surface model; (2) geological field survey; detection of major faults and joints, and (3) the identification of fault zones with the combination of UAV, TLS, and structural geological analysis including the visualisation of faults in the 3D surface model.

On the active parts of the quarry, terrestrial laser scanning measurements (TLS) were collected that further provided high accuracy and high-resolution reference data. Due to extensive ongoing work, we did the first set of TLS measurements on the same day as we carried out the UAV flights, while the second TLS measurements took place three days later. The TLS produced ultra-high-density point cloud (6 mm resolution on 10 m per station) with high accuracy; however, huge rocks and limited ground accessibility caused remarkable occlusions in the TLS point cloud. We used a phase-based terrestrial laser scanner (Faro Focus 120S—manufactured by FARO Technologies, Inc., Lake Mary, FL, USA) with +/−2 mm ranging accuracy and 120 m maximum measurement range. All the quarry levels had been surveyed by TLS; 19 stations on the first, 31 on the second, 42 on the third, and six on the fourth level (Table 1). The point clouds of each level had been merged by an ICP (iterative closest points) algorithm (matching error below 8 mm for each level) while the unified point cloud of the levels had been georeferenced by the GNSS coordinates of the GCPs (residuals: below X: 1.8 cm; Y: 1.5 cm; Z: 2.4 cm for each level). The measurement range enabled to scan reasonable areas from a particular level to an upper/lower one. Therefore, the quality of the point cloud matching was checked by the comparison of the overlapping areas. For validating the UAV point cloud whether it can be used for engineering geological analysis, its geometric accuracy and correctness are to be assessed. However, it is necessary to compare the displacement and rotation of the two point clouds, namely the one of UAV and that of TLS. The TLS point cloud with its highest point density and measurement accuracy serve as a reference for comparison. Selected parts (containing characteristic, easy-to-identify points) of the TLS point cloud have been compared to that of the UAV point cloud and used as a reference to this study. Spatial distance measurements have been carried out at the large 40 × 40 cm size plastic targets that can be well identified in both point clouds (used also as GCPs exclusively for the UAV point cloud) on the first quarry level and the mean discrepancy was found to be below 3 cm (Table 2). The comparison proved that the UAV-based point cloud fit well to the TLS point cloud and therefore is able to support further geological analysis. Then the two point clouds were merged and resampled for further processing.

The UAV survey was carried out on the 12 October 2018, under good weather conditions (sunny, moderate wind speed). The applied system is a commercial DJI Phantom 4 Pro equipped with DJI’s camera FC6310_8.8 (focal length: 8.8 mm) that is capable of capturing 5472 × 3648 (RGB) resolution images (sensor dimensions: 12.833 × 8.556 mm) [50]. 

**Figure 4:**
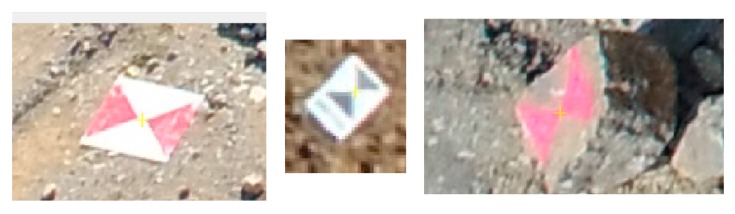
Three types of the GCPs, images taken from the UAV.

A Leica CS10 GNSS receiver was used with a Gs08plus antenna to measure the ground control points (GCPs) that allow cm-accuracy. For the georeferencing, in addition to small, 20 × 30 cm and large, 40 × 40 cm size plastic targets, several points marked with luminous red colour on the surfaces of the rocks were used as GCPs (Figure 4). These 25 GCPs were evenly distributed around the mapped area. The measurements mean spatial accuracy was 2.5 cm (minimal value: 1.5 cm, maximal value: 7.5 cm).

**Figure 5:**
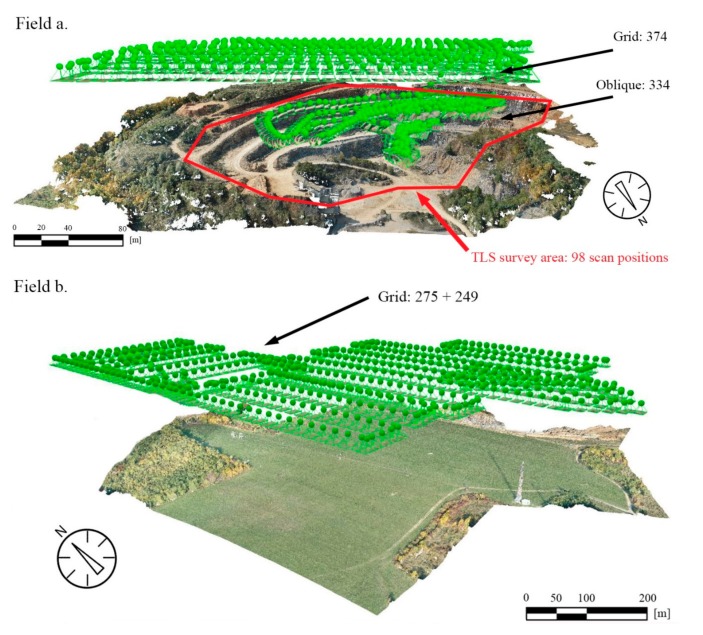
UAV image locations with numbers of grid plus oblique images and TLS survey area at the quarry: ‘Field a’ (**top**); UAV image locations and number of grid images at the plateau: ‘Field b’(**bottom**).

Due to the size and fragmentation of the area to be surveyed, the survey was done in two parts: the quarry (Field a) and the plateau (Field b). Four flights covered the entire area to be mapped. The main parameters of the four flights and the two pixel-based reconstructions are given in Table 3. The quarry face is intensively dissected, and it has several vertical and sub-vertical walls. Therefore, one of the flights in the quarry was controlled manually with an oblique camera angle, supported by the first-person view function of the device. The speed was limited to 2 m/s with autofocus with varying gimbal angles. This flight for the quarry area (Field a), produced 334 images (20 Mpixel each)of which 141 were georeferenced in WGS84 reference system. Orientation angles have also been embedded in the Exif, which could be used for preliminary image registration. The other three flights captured the area of the quarry in the ‘Field a’ area and the parts that are to be involved in further mining procedures in the ‘Field b’ area. Prior to these surveys, flight planning was carried out. In order to achieve high-quality image datasets, the overlap between rows was ensured to be above 75%, while that of between consecutive images was above 80%. For these autonomous flights, waypoints were given [51] with the camera set to nadir direction, and autofocus was applied due to varying altitude; the flights provided 374, 275, and 249 georeferenced images as a result (Figure 5).

**Figure 6:**
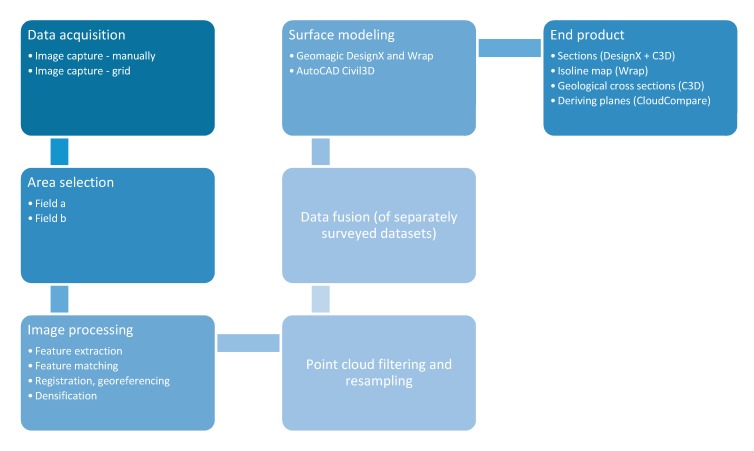
Major steps of data processing from UAV and TLS to surface modelling and geological applications.

The major steps of UAV data acquisition and the processing of the end products that were used in engineering geological applications are given in Figure 6.

As the first step in preparing SfM (structure from motion) based data processing, the captured images are to be investigated [3]. During the visual check, the bad (low quality, blurred, burned) images should be filtered out, and the corresponding image sets should be selected. The applied software automatically extracts the key points and the relevant image pairs based on the prepared dataset. The relative orientation is based on the tie points and the photo capture locations. The result is a sparse point cloud containing the tie points. The GCPs are to be manually selected in the dataset that can be supported by the processing software, once the GCP is selected in two corresponding images. Then, the point cloud can be densified to be compared with the TLS-acquired one.

The surface model was created in Geomagic DesignX. Since it contains multiple errors (e.g. holes, gaps, fragmented elements), these have been corrected either manually or using semi-automatic methods (in Geomagic Studio).

**Figure 7:**
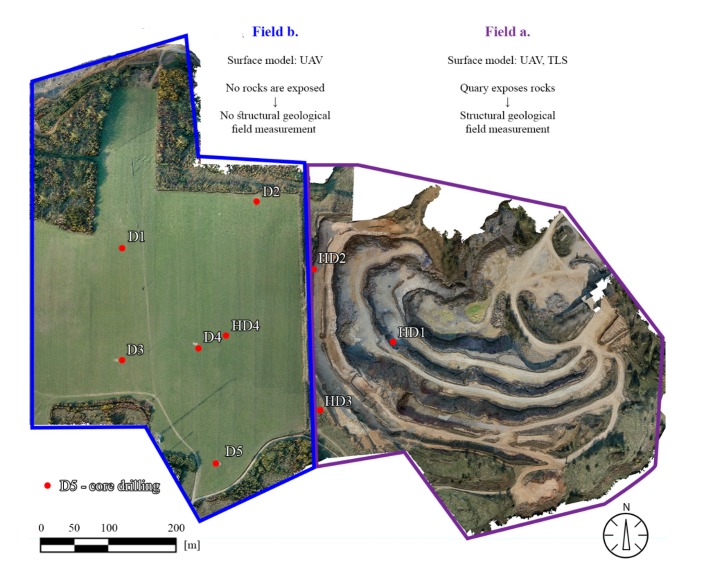
Surface model of the area: Field a (quarry), where both TLS and UAV were used, and due to exposed rocks it was possible to measure structural geological elements on-site, while at Field b (plateau) where only UAV was used to generate the 3D surface model it was not possible to measure structural geological elements (faults and bedding planes) with geological compass. The location of former core drillings (HD) and more recent ones (D) are also shown.

The resulted high-density, photorealistic model enables the derivation of further products; it contains in total 11,472,014 vertices (22,908,994 polygon faces) and covers 0.661 km^2^. The RGB data enable to obtain photo-realistic surface models (Figure 7).

For the geological reserve calculations, the area of interest (almost 0.2 km^2^) has been cropped and uniformly resampled; from the ~10,000 points kept a TIN model with ~20,000 triangles has been derived. Reducing the number of points is required to work in a CAD environment; the final set of 10,090 points (extracted from the surface model) described the particular area topography properly (number of triangles: 20,045, 3D surface area 0.197 km^2^, maximum triangle length: 42.87 m, minimum triangle length: 0.039 m).

Besides the surface model created in Civil3D, the locations of the faults were also presumed based on the information from the lithological descriptions of previous boreholes. A model was created connecting locations of field measurements with dip and direction data. The obtained data were then integrated and illustrated on the surface model, locating the main fault zones. This way the geological formations can be represented as wireframe models. The resulting planes were checked and compared to major structures shown on formerly published geological maps (Figure 3). The fault planes that were identified and confirmed this way have been drawn in the surface model. These onsite measurement values have been compared to those derived only from the dataset with the CloudCompare “Compass” plugin. The results show a good correlation; the plane parameters can be derived from the high density, coloured surface model, too.

A data set of previous core drillings (HD) and more recent core drilling (D) were gathered. The geological descriptions of the drillings were evaluated. The locations of the drillings were identified according to their coordinates, and they are shown on the 3D field model (Figure 7).

**Figure 8:**
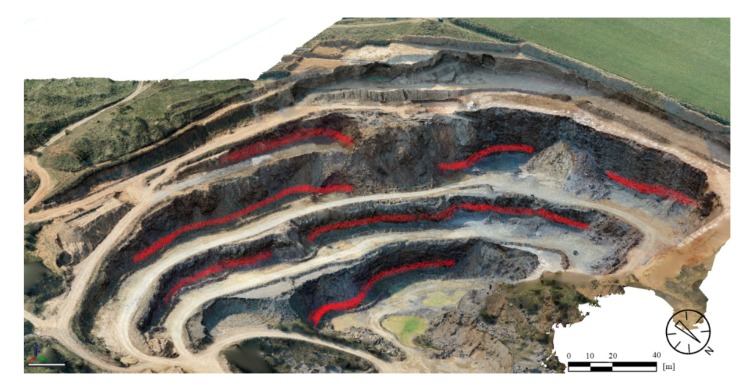
Parts of quarry faces where field structural geological measurements were possible (red markings). All other parts were inaccessible, and TLS and UAV were used to identify structural elements such as fault planes.

The geological field survey focused on the description of major lithotypes and on the identification of major geological units. Structural geological field measurements aimed to recognise faults and joints and bedding planes. It was only possible to detect structural geological data in the quarry area, since at the plateau, no rocks were exposed (see Figure 7). Due to the steep walls and to safety regulations, it was not possible to access most parts of the exposed walls. However, during the field survey, 322 structural elements were measured in the quarry. Only at the lowermost two meters of rock faces were accessible; therefore, only field measurements provided data for those parts (Figure 8). These included bedding planes, joints and faults. Dip direction and dip angle pairs were recorded with geological compass or the RockLogger application depending on which measuring technique was suited better to the orientation of the surface of the discontinuities. Recorded data were plotted with two-dimensional stereographic projection using the Rocscience Dip software.

**Figure 9:**
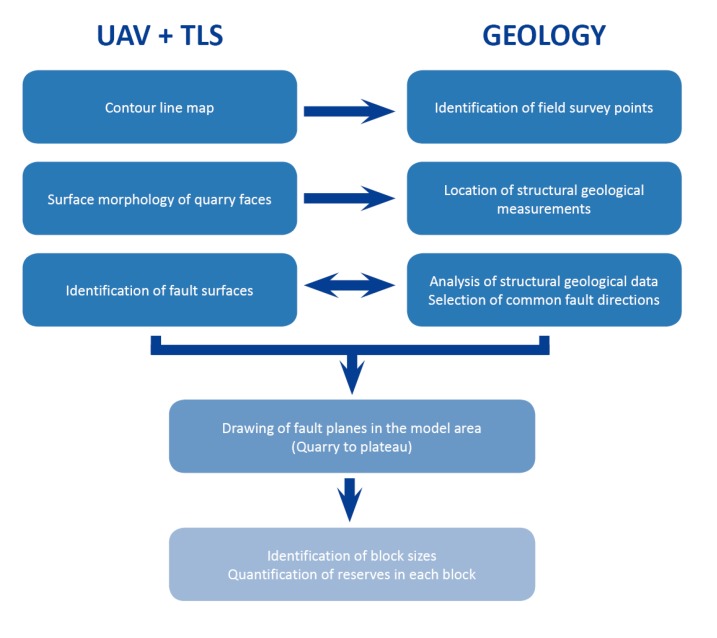
Application of UAV and TLS in structural geological measurements in this paper.

The combined data set was used in the structural geological survey. In the created contour line map and images as well as the quarry surfaces, the major fault zones were identified. These zones were surveyed in the field, and also field observations were compared to the UAV and TLS generated surface model. The most common structural geological directions were analysed in the data set, and the major fault zones were identified. These structures were imported into the model and visualised as major structural geological planes of the quarry and the plateau zone. Reserve calculations were made using Civil3D software (Figure 9). The surface of each lithological unit was drawn based on field data and core data. The volume of cover beds and reserve of limestone deposit was calculated for five blocks. In each block, the lower and upper surface of the lithotypes (lithological units) was selected, as well as the delineating faults. The volume between the depicted surfaces was computed using the volume calculation command of the Civil3D.

## 4. Results

**Figure 10:**
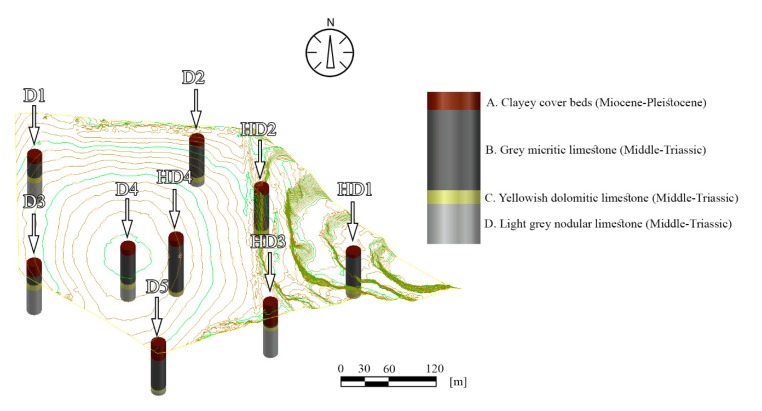
3D topography of the area showing previous core drillings (HD) and recent core drilling (D), The idealised lithologic column of Triassic limestone reserve (B-D) and cover beds (A) are also shown. The lithologic units (A-D) representing different qualities were used in the reserve calculations.

The high-resolution surface model derived from the UAV and TLS allowed the acquisition of several end products, for example, views and sections or (e.g., isoline) elevation maps such as contour line maps. On these maps, the locations of archive core drillings were also identified. The idealised lithological column was also complied based on quarry observations, field survey, and archive data of core drillings (Figure 10). Miocene–Pliocene clayey cover beds represent the youngest geological formation below the surficial soil layers (lithologic unit A). These clayey beds are considered as waste material. Below these layers, a Middle-Triassic high quality, carbonate-rich grey micritic limestone (lithologic unit B) is found (Figure 10). It is underlain by yellowish dolomitic limestone (lithologic unit C) which is the lowest quality carbonate reserve material due to its dolomite content. The lowermost beds are represented by light grey nodular limestone (lithologic unit D) of Middle-Triassic period. These beds of lithologic unit D are considered as medium quality limestone reserve since a minor amount of clay is also found in these types of beds [39].

**Figure 11:**
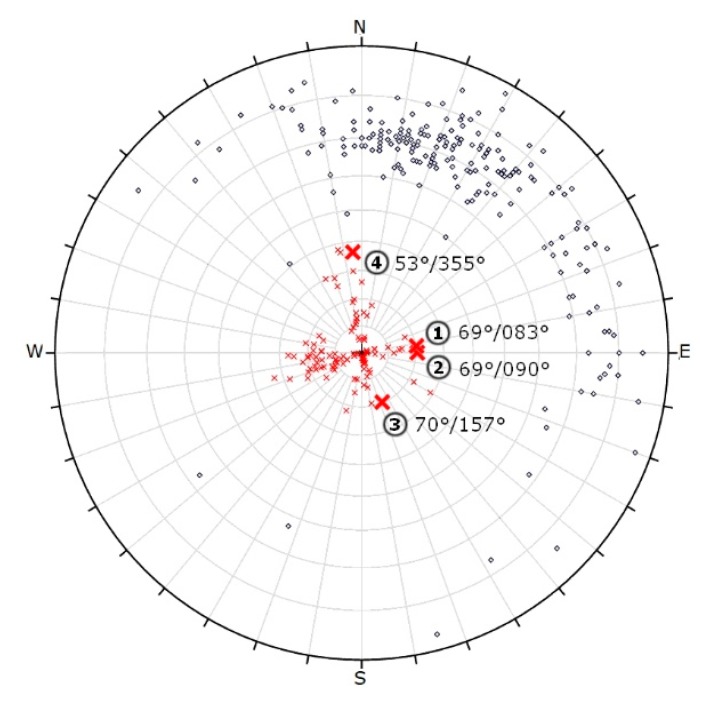
Stereographic projection of discontinuities using equal angle projection to the lower hemisphere with dip vectors and polar stereonet (x ~ fault, o ~ bedding). The four major faults are numbered: 1–4.

**Figure 12:**
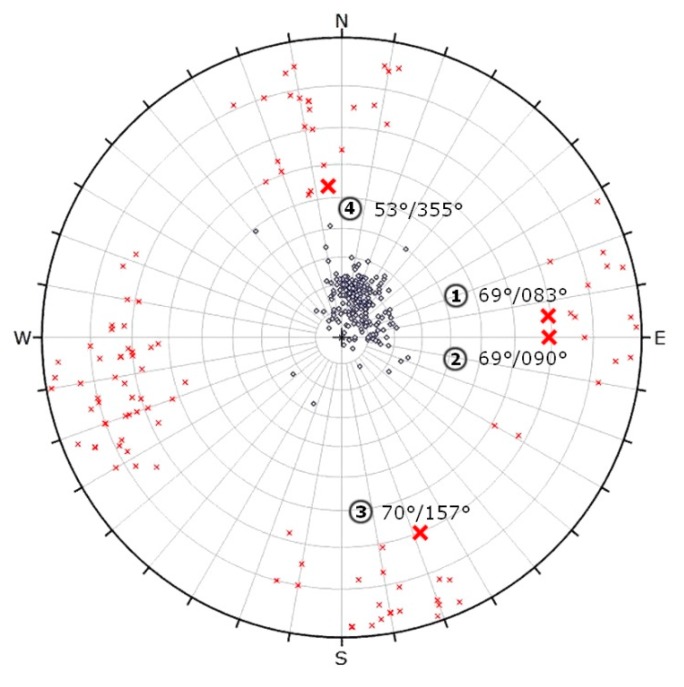
Stereographic projection of discontinuities using equal angle projection to the upper hemisphere with pole vectors and polar stereonet with highlight of the four major faults (x ~ fault, o ~ bedding). The four major faults are numbered: 1–4.

From a structural geological point of view, the field data set provided a reference and control for the fault zones. Stereographic projections of all measured discontinuities of the quarry with two different methods are given (Figure 11 and Figure 12). The orientation data uses dip vectors which are more capable of representing the deviation of the less steep-angled joints (Figure 11). When the same joint sets are plotted using pole vectors, the highlight of the four major faults is possible (Figure 12). The pole plot is suitable for analysing the joints with steep dip angles. Based on these results, it can be seen that the dip angle of the bedding planes is consecutively lower than the dip angle of the faults. The dip of the layers is typically between 5° and 25°, while the dip angle of the fault is mostly above 60°. It can be observed that the dip directions (measured from North by right-handed) of the recorded bedding planes have a high deviation, but at the same time, they typically point to the direction between 340° and 100°, to North-Northeast. The dip directions of the faults are different, as they point to four perpendicular directions to each other: 80°, 170°, 260°, 350°.

**Figure 13:**
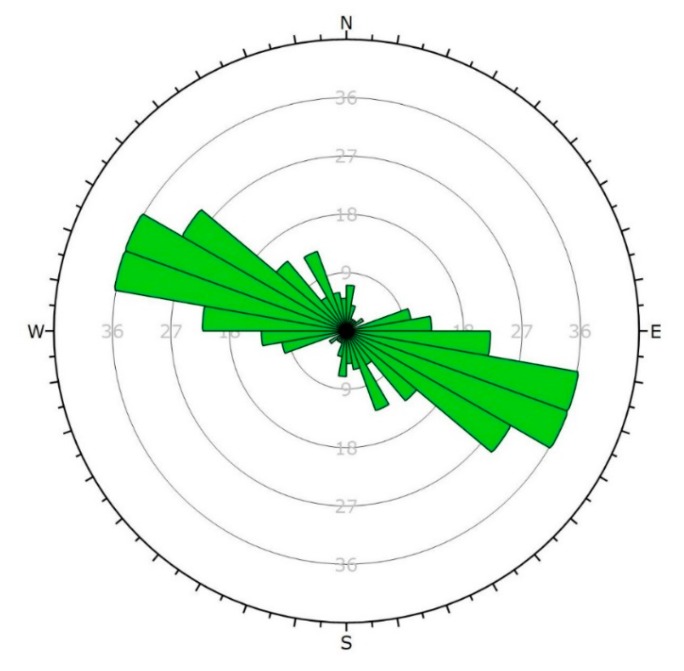
Rosette plot of bedding planes.

**Figure 14:**
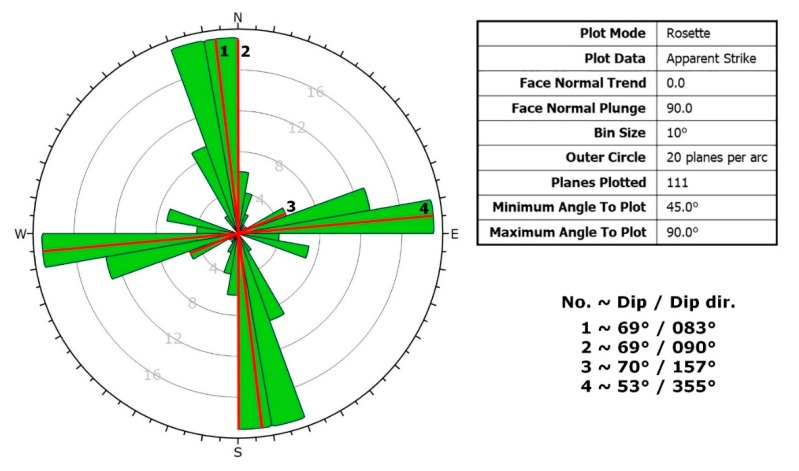
Rosette plot of fault planes with highlight of the four major faults (numbered by 1–4).

**Figure 15:**
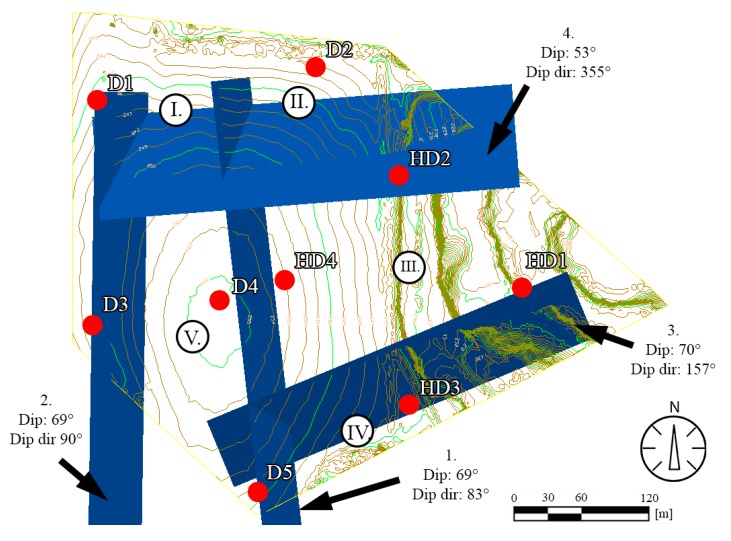
Major identified fault planes dissecting the contour lines (major faults are numbered 1–4) as seen from W. Blocks bordered by major faults and used for reserve calculations are numbered by I–V.

The bedding and fault planes are also plotted in rosette plots (Figure 13 and Figure 14). This plot type shows a radial frequency histogram of the strikes of the joints; in other words, it represents the density of the strikes. The frequency histograms demonstrate the frequency of values that fall into equal-sized intervals (bins). Thus, the rosette plot diagrams show the number of strikes that fall into a given interval (in this case it is 10°). By means of this method, the characteristic strikes can be identified visually. Indirectly, the same information can be obtained about the dip directions. The strikes of the bedding planes (Figure 15) are most typically in the range of 100–120°; therefore, the most frequent dip directions in case of the beddings fall into the range 10–30°. In case of the faults (Figure 14), the four above-mentioned perpendicular directions can be observed again, from which the strike in the direction of 170–350° proved to be dominant.

The location of major fault planes was determined from and UAV-TLS surface model. The geological information obtained from former boreholes were also considered and with the combination of these methods: the field survey, the remote sensing and archive core descriptions, and the major fault zones were localised on the contour map of the area (Figure 15). Taking into account the block geometries, the fault bonded major reserve blocks were also depicted: blocks I–V (Figure 15).

**Figure 16:**
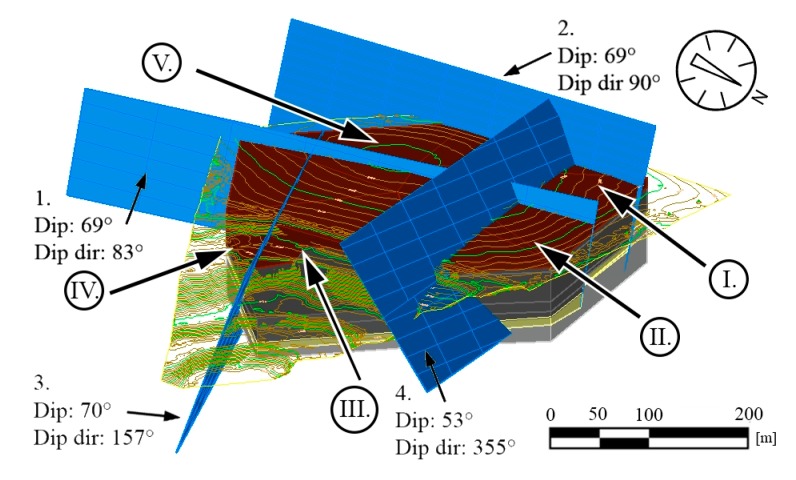
Simplified 3D model of the area with identified major faults and rock beds. The displacement of faults was compiled based on field data and core drillings. The figure also shows the five blocks (numbered by I–V) that are bordered by major faults and used for reserve calculations.

Reserve calculations were made for five blocks (block no: I–V). Four lithological units were considered: A, clayey cover beds (waste material); B, grey micritic limestone (highest quality limestone reserve); C, yellowish dolomitic limestone (poor quality limestone reserve), and D, light grey nodular limestone (medium quality limestone reserve) (cf. Figure 10). The major faults and their displacements were also considered in the calculations, and a 3D model was generated showing major faults (Figure 16). Fault planes were considered as plane surfaces, and no bending or undulations were considered.

**Figure 17:**
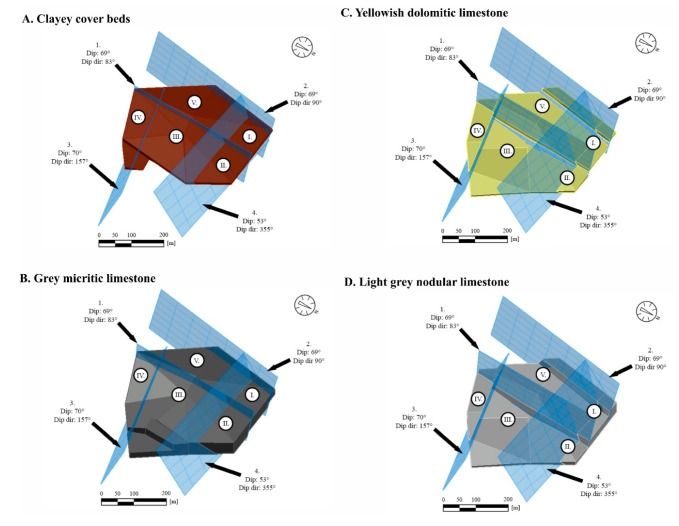
(**A**) Clayey cover beds (waste material), (**B**) Grey micritic limestone (highest quality limestone reserve), (**C**) Yellowish dolomitic limestone layers (poor quality limestone reserve) and (**D**) Light grey nodular limestone deposit (medium quality limestone reserve) in blocks I–V that are bounded by faults 1–4 (the reserves were calculated for each block).

For each lithological unit (A–D), a 3D model was generated showing the major fault systems, numbered 1–4, that delineates each block (Figure 17A–D). 

**Figure 18:**
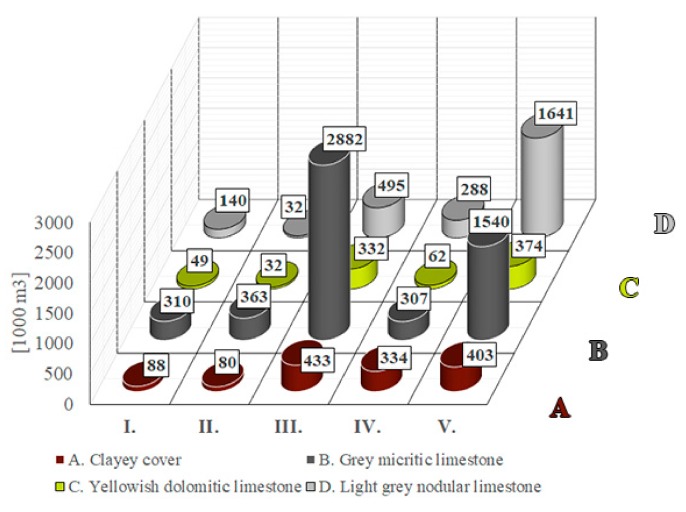
Calculated reserves in 1000 cubic metres of main lithological units (A–D) in blocks I–V (locations of the blocks are given in Figure 12 and Figure 17and for lithological units and fault boundaries see Figure 16). Clayey cover beds (unit A) are waste material, while unit B has the highest quality limestone (see text for further explanation).

According to the calculations, the clayey cover beds (unit A, quarry waste material) has a total volume of 1,338,000 m^3^, which is relatively evenly distributed with the exception of block I and II where fewer cover beds are found (Figure 18). The largest reserve is available for exploitation from the high-quality grey micritic limestone (unit B) with a total volume of 5,402,000 m^3^. This reserve is unevenly distributed since major parts of it are allocated in blocks III and V (Figure 18). With respect to the poorest quality limestone reserve, the yellowish dolomitic limestone, (unit C), the area has a reserve that is less than 1 million m^3^ (namely 849,000 m^3^). The lowermost bed, the light grey nodular limestone (unit D, medium quality limestone reserve), has the second-largest reserve with a total volume of 2,596,000 m^3^, but this reserve is mostly found in block V (Figure 18).

## 5. Discussion

In areas where the quarry faces are not visible from the ground level, it is necessary to use UAV [33]. The combination UAV and TLS methods could bring the required resolution and enable to create a surface model [28,29], but our study shows that it is also crucial to identify faults and draw fault planes, on the 3D terrain model, in areas where no direct surface information on structural geology is available (Figure 15). 

At first, the UAV flights that were needed for the surface model is discussed. The UAV flight mode is most commonly a programmed itinerary in case of large flat surfaces like Field b. This method is often used when larger areas are mapped with no oblique or nearly vertical cliff faces [38]. In this study, the pre-programmed itinerary flights in grids were used in the plateau area (Figure 5, bottom part ‘Field b’), but for the quarry face detection, the programmed grid flights did not bring enough information and thus additional manually controlled flights were also necessary. During these flights, oblique images were taken that mapped the steep and dissected quarry face (Figure 5, top part ‘Field a’). The combination of these two types of flights provided appropriate accuracy and data set (Table 1). The obtained data set was used to create a photo-realistic surface model (Figure 7). However, it is necessary to analyse the plane surfaces of the surface model to identify the presence of faults. There are several methods to select structural geological elements from a large amount of digital data [52], but we have used UAV and TLS data set. It has been emphasised in previous works that a field control is essential in the structural geological interpretation and remote sensing techniques alone might not provide enough data on fault and fold system [43,53]. In this study, it was possible to measure bedding planes and faults only at the lowermost parts of quarry faces (Figure 8), but the upper parts were inaccessible. At upper parts of the quarry, the possible fault planes were selected from the surface model. The most common surfaces were depicted, however these surfaces might not be the ones that caused major fault displacements [43]. 

The field measurements also allow the detection of fault directions but selecting faults that cause major replacements require additional analyses [53]. The lithological logs of core drillings could provide data for the presence of major faults (Figure 10). Nevertheless, the field control of such faults is necessary, and the faults identified in the quarry can be linked to the core data (Figure 11). The direction of major faults can be controlled by field measurements (Figure 12 and Figure 14). Where no outcrops or exposures are available, and it is not possible to measure faults in the field (‘Field b’ on Figure 7), it is necessary to project the recorded fault planes of the neighbouring area. We have used this method to draw the fault planes of the plateau area (Figure 15). By detecting and outlining faults on unexposed and exposed areas, it is possible to identify major operational units (blocks) of quarrying (Figure 16). It has also been outlined earlier that it is beneficial to divide the area into smaller units, blocks [33]. The blocks provide a base for reserve calculations. 

For obtaining the volumes of reserves, a 3D model is required [32]. The reserve calculations are simpler when there are uniform rock beds such as the ones in a marble quarry [33]. In our case, four lithological units were identified (Figure 10), and they represent different qualities. It was necessary to give the thickness of each lithological unit within the blocks, which was possible based on the data of core logs (Figure 17). With the help of this subdivision, it was possible to calculate the reserves of each lithological unit (A–D representing different qualities) by Civil3D (Figure 18). With this software, the block sizes can be measured, and reserves of each block can be calculated. The reserve calculation allowed the identification of the blocks where a large volume of high-quality limestone (unit B) was found: in block III and block V (Figure 18). This also allows for the calculation of the amount of waste materials (cover bed, unit A), that has to be removed during the quarry operation of each block (Figure 18). The accuracy of such calculations is in the order of cubic meters (or even higher) that provide valuable information on existing and exploitable reserves. On the other hand, the accuracy of the reserve calculations could be less, when the faults are curved or undulating and do not form plane surfaces. The obtained structural geological data allow the planning of quarry operations: identification of high-quality reserves, cover beds and the calculation of the volumes of reserves in between fault zones (Figure 16 and Figure 18). When a more detailed lithological description is available, it is possible to refine reserves, and if high-quality core description is obtainable, a further subdivision of lithological units and more detailed reserve calculations are feasible in the future. In summary, this enables the sustainable exploitation of raw materials and feasible management of quarrying activities.

## 6. Conclusions

The combination of UAV with TLS provided a solid background to generate a 3D surface model of the quarry area and a vegetation-covered plateau. The UAV flight itinerary was programmed for the plateau area providing a dataset in a grid system, while for the dissected quarry face, both programmed itinerary flights and manually controlled oblique imagery were used. The TLS was only applied in the quarry. Our experience demonstrates that without manual control and oblique images, it is not possible to map irregular, often steep and fault dissected quarry faces. The accuracy (below 10 cm) and spatial resolution (5 cm point spacing in the resampled dataset) of the resulted point clouds enable us to create a digital surface model appropriate to geological analysis and for reserve calculations. The detection of major faults was made at the lowermost two meters of the quarry faces in the field, but these field measurements do not provide enough data for structural geological interpretation and for the selection of major faults. Hence, a surface model of the quarry face obtained by UAV and TLS was also used to identify major fault zones. Historic core drilling descriptions were collected to identify major lithological units, but this data set alone does not provide enough data to identify major faults. Accordingly, a combination of remote sensing technique, structural geological field measurements, and lithological core logs were needed to localise and identify major fault zones. A 3D surface model with the fault planes allowed us to divide the area into five major blocks. The reserve calculations of these blocks were possible based on the modelling of the four identified lithological units, representing waste material (cover beds) and low- to high-quality limestone reserves. This paper demonstrates that the application of combined methods (field survey, archive core data, and remote sensing) provide all essential data for the calculation of raw material reserves and support the sustainable operation of quarries by minimising the quantity of damp material.

## Figures and Tables

**Table 1:** Terrestrial laser scanning parameters.

-	Faro Focus 120 Parameters
ranging technique	phase-based
ranging accuracy	+/− 2 mm
applied point spacing	6 mm @ 10 m
number of scan positions	98
number of scanned points	588 M
quarry area covered	0.15 km^2^

**Table 2:** Comparative assessment of UAV point cloud vs TLS point clouds—discrepancies at GCPs.

-	X [mm]	Y [mm]	Z [mm]
GCP 1	–0.5	–20.1	0.1
GCP 2	8.0	–19.5	22.1
GCP 3	–22.6	–19.5	28.7
GCP 4	–22.5	18.8	–13.1

**Table 3:** Flight and image processing information.

-	Field a (Quarry Area)		Field b (Plateau Area)	
Sensor Parameters	focal length: 8.8 mm, sensor size: 12.833 × 8.556 mm			
Image Size	5472 × 3648 (RGB)			
Flight	I	II	III	IV
Flight Mode	Oblique	Grid	Grid	Grid
Height	Various	140.5	120.9 m	120.9 m
Length	-	5.1 km	4.4 km	3.3 km
Image Count	334	374	275	249
Overlap between Images	various	>75% (between rows), >80% (between consecutive images)		
Area Covered	0.256 km^2^		0.654 km^2^	
Average GSD	2.57 cm		3.15 cm	
Used GCP	14		14	
GCP’s RMS (x,y,z)	2.0, 1.9, 2.7 cm		1.2, 1.1, 7.0 cm	
Mean Number of 2D Keypoint	23,058		29,480	
Mean Number of 3D Tiepoint	6,462		7,557	
Mean Reprojection Error	0.12 pixel		0.13 pixel	
Image Scale for Densification	1/1		½	
Number of 3D Densified Points	320,727,823		58,551,776	
Average Density	970.47		129.22	
